# Retraction Note: Meta-analysis of the efficacy of pancreatoduodenectomy with extended lymphadenectomy in the treatment of pancreatic cancer

**DOI:** 10.1186/s12957-015-0547-0

**Published:** 2015-03-26

**Authors:** Xinbao Xu, Hui Zhang, Ping Zhou, Lei Chen

**Affiliations:** Department of Hepatobiliary Surgery, Airforce General Hospital of Chinese People’s Liberation Army, Beijing, 100142 China; Department of Radiotherapy, Airforce General Hospital of Chinese People’s Liberation Army, Beijing, 100142 China; Department of Hepatobiliary Surgery, Peking University People’s Hospital, Beijing, 100044 China

## Retraction

The Publisher and Editor regretfully retract this article [1] because the peer-review process was inappropriately influenced and compromised. As a result, the scientific integrity of the article cannot be guaranteed. A systematic and detailed investigation suggests that a third party was involved in supplying fabricated details of potential peer reviewers for a large number of manuscripts submitted to different journals. In accordance with recommendations from COPE we have retracted all affected published articles, including this one. It was not possible to determine beyond doubt that the authors of this particular article were aware of any third party attempts to manipulate peer review of their manuscript.

## References

[1] Xu X, Zhang H, Zhou P, Chen L (2013). Meta-analysis of the efficacy of pancreatoduodenectomy with extended lymphadenectomy in the treatment of pancreatic cancer. World J Surg Oncol.

